# Comparison of two minimally invasive surgical approaches for hypertensive intracerebral hemorrhage: a study based on postoperative intracranial pressure parameters

**DOI:** 10.1186/s12893-023-02306-x

**Published:** 2024-01-03

**Authors:** Minxue Lian, Xiaolei Li, Yuangang Wang, Hongmin Che, Zhongnan Yan

**Affiliations:** 1 Department of Neurosurgery, Xi’an Gaoxin Hospital, 16 Tuanjie South Road, Xi’an, Shaanxi Province 710061 China; 2 Department of Neurosurgery, the first Affiliated Hospital of Xi’an Jiaotong University, Xi’an, Shaanxi China

**Keywords:** Hypertensive intracerebral Hemorrhage, Intracranial pressure, Transsylvian insular approach, Transcortical approach, The length of hospitalization, Postoperative consciousness recovery time

## Abstract

**Background:**

Increased intracranial pressure (ICP) in patients with hypertensive intracerebral hemorrhage (HICH) has been associated with poor prognosis. The transsylvian insular approach (TIA) and the transcortical (TCA) approach are applied for patients with HICH. We aimed to compare the postoperative ICP parameters of TIA and TCA to identify which procedure yields better short-term outcomes in patients with basal ganglia hematoma volumes ranging from 30 to 50 mL.

**Methods:**

Eighty patients with basal ganglia hematomas 30–50 mL were enrolled in this study. Patients were implanted with ICP probes and divided into TIA and TCA groups according to the procedure. The ICP values were continuously recorded for five days at four-hour intervals. Short-term outcomes were evaluated using the length of hospitalization and postoperative consciousness recovery time.

**Results:**

No statistically significant differences were found in age, sex, GCS score at admission, hematoma volume, and hematoma clearance rate (p > 0.05). The results showed that postoperative initial ICP, ICP on the first postoperative day, mean ICP, DICP20 mmHg × 4 h, postoperative consciousness recovery time, the length of hospitalization, mannitol utilization rate and the mannitol dosage were lower in the TIA group than in the TCA group (p < 0.05). Postoperative consciousness recovery time was positively correlated with ICP on the first postoperative day, and the length of hospitalization was positively correlated with mean ICP.

**Conclusions:**

TIA is more effective than TCA in improving the short-term outcomes of patients with basal ganglia hematoma volumes ranging from 30 to 50 mL according to comparisons of postoperative ICP parameters.

## Introduction

Hypertensive intracerebral hemorrhage (HICH) is a common and serious cerebrovascular disease that occurs during neurosurgery and is characterized by an acute onset, high mortality, high disability, and high morbidity. It frequently occurs in middle-aged and older individuals, with peak incidence in winter and spring. Recently, long-term follow ups have indicated that the disability and mortality rates are increasing [1]. Additionally, the age-at-onset of HICH has been decreasing among patients, placing a heavy burden on the families of patients affected by HICH [2].

Two options are feasible according to the condition of patients with HICH and the amount and location of hematomas: conservative treatment and surgical evacuation. Theoretically, surgical evacuation effectively reduces the mass effect of the hematoma, relieves intracranial hypertension and mechanical compression, facilitates the survival of the penumbra in functionally impaired areas, and improves prognosis. Surgical methods include conventional craniotomies, stereotactic punctures, and minimally invasive craniotomy [3–6]. The minimally invasive surgery concept has been widely used in neurosurgery to treat patients with HICH who have a hematoma volume of 30–50 mL and mild disturbances of consciousness because it reduces surgical trauma and achieves excellent therapeutic efficacy [7, 8]. The commonly used minimally invasive surgical approaches for patients with HICH include the transsylvian insular approach (TIA) and the transcortical approach (TCA); however, the resultant trauma to normal brain tissue differs between the two approaches. The protocol for TCA-based surgery is relatively simple, and the operation time is short. However, the cerebral cortex gets greatly damaged owing to the continuous pulling of the cerebral cortex to provide surgical space during surgery, resulting in possible damage to the functional cortex as well, exerting adverse effects on the prognosis. The TIA-based surgery is a complex procedure characterized by extensive duration. It effectively accesses the hematoma cavity through the brain’s natural space, facilitating cerebrospinal fluid release, and efficiently clearing the central area of the hematoma within a short timeframe. This approach expands the surgical field of view, minimizing the risk of overlooking surrounding hematomas, thereby enhancing hematoma clearance rates and promoting the postoperative elimination of brain edema. In addition, TIA results in a shorter trajectory and obviates the need for cerebral cortex manipulation, thereby minimizing brain tissue damage and promoting postoperative recovery of cerebral nerves [9, 10]. The use of TIA for the removal of basilar ganglia hematomas was first documented in 1972. Via an inherent fissure between the frontal and temporal lobes, as well as the insular lobe, the TIA allows access to the underlying basal ganglia. TIA also offers a shorter distance to the hematoma cavity, more precise localization, and clearer visualization, than does TCA, thus facilitating hematoma removal and accurate hemostasis [11].

Increased intracranial pressure (ICP) is generally observed in patients with HICH and has been consistently associated with poor neurological outcomes, which are the leading causes of high mortality in patients with HICH [12]. The morbidity rate of intracranial hypertension in patients with HICH is 67%, whereas that caused by intracranial hypertension is 50% [13]. ICP monitoring has been widely applied in the clinical treatment of HICH, and ICP parameters such as pressure reactivity index (PRx), dose of ICP (DICP), regression of amplitude and pressure (RAP), and cerebral perfusion pressure (CPP) have been utilized to predict the prognosis of HICH [12, 14]. Furthermore, the frequency of ICP values higher than 20 mmHg, ICP variability, and mean ICP are independently correlated with mortality and prognosis in patients with HICH [15]. In addition, dynamic monitoring of ICP can guide drug treatment, surgical treatment, and postoperative management of patients with HICH to improve the survival rate and prognosis; however, reports on continuous ICP monitoring after surgery for HICH are lacking, and few studies have reported on the efficacy of monitoring of the TIA or TCA in patients with HICH. Both TIA and TCA have proven to be effective and safe for the treatment of HICH when the basal ganglia hematoma volume ranges from 30 to 50 mL. Therefore, we aimed to compare the postoperative ICP parameters of TIA and TCA to identify which procedure yields better short-term outcomes in patients with basal ganglia hematoma volumes ranging from 30 to 50 mL.

## Materials and methods

### Patients and eligibility

We retrospectively analyzed the hospital records of 80 patients with HICH after neurosurgery and admission to Xi’an Gaoxin Hospital between January 2019 and December 2022; these patients included 48 males and 32 females aged 42–68 years with different degrees of neurological dysfunction, such as aphasia and limb movement disorders, among others, with GCS scores ranging from 4 to 12 on admission. All patients were treated with minimally invasive surgical approaches and divided into TIA and TCA groups according to the surgical approaches, which were performed by the same team of neurosurgeons with extensive surgical experience and randomly selected by the patients’ legal representatives. The study protocol was approved by the Xi’an Gaoxin Hospital Ethics Board.

The inclusion criteria were (1) medical history of hypertension; (2) basal ganglia hematoma (amount 30–50 mL) confirmed by CT; (3) postoperative implantation of an ICP probe > = 5 days; (4) absence of intraventricular hemorrhage; (5) disease onset within 6 h and GCS score ranging 4–12 at admission; and (6) no observed preoperative brain herniations. The exclusion criteria were (1) any underlying disease, including other cardiovascular and cerebrovascular diseases, liver or kidney dysfunction, coagulation disorders, or intracranial infections, affecting the prognosis; (2) hemorrhage due to tumors, trauma, coagulopathy, aneurysms, or arteriovenous malformations and hemorrhage after infarction or long-term use of antiplatelet or anticoagulant drugs; (3) death within 5 days of surgery; and (4) recurrent hemorrhages necessitating surgery. The inclusion and exclusion criteria were the same for both groups.

### Surgical methods and postoperative treatment

In our study, intracranial hematomas were evacuated via TIA or TSA using microscopy for patients with HICH carrying basal ganglia hematoma (30–50 mL) after informed consent was obtained from the patients’ legal representatives for surgical treatment. Within the hairline, a 6 cm incision (straight or curved) was made according to the location of the intracranial hematoma and the surgical approach, and the window was approximately 3 cm × 4 cm. For TCA, a small incision was made in the frontal or temporal cortex with fewer vascular and non-functional areas closest to the intracranial hematoma. For TIA, after the Sylvian fissure was split along the frontal lobe side and the insular cortex was located, a small incision was made parallel to the Sylvian fissure. After entering the hematoma cavity along the incision using microscopy, the hematoma was evacuated using a suction device and continuous irrigation. Electrocoagulation for hemostasis was performed on the sources of persistent bleeding because small hematomas may not be completely evacuated when blood clots adhere tightly to the surrounding neurovascular structures. After the hematoma evacuation, the hemostatic fibrils were filled, and an ICP probe (ICP MicroSensor, SOPHYSA France, REF: PSO-PT) was implanted in the hematoma cavity.

When the ICP value was higher than 20 mmHg, we administered 125 mL mannitol every 12 h; when found ineffective, 125 mL furosemide was administered every 8 h. When the postoperative ICP value was higher than 25 mmHg, 125 mL mannitol was administered every 6 h in combination with 20 mg furosemide or 20 g human blood albumin twice a day. In cases of sudden and sharp increase in ICP values, besides changing the body position, strengthening the nursing care, and lowering the ICP with drugs, we urgently checked the head CT to clarify the intracranial hematoma and excluded the possibility of re-hemorrhage.

The ICP values were monitored for 5 days because of the risk of intracranial infection and the instructions and requirements of the ICP probe. All the patients were treated according to the guidelines for HICH management.

### Data collection

The following data were recorded for all enrolled patients with HICH: age, sex, GCS score at admission, hematoma volume, hematoma clearance, length of hospitalization, and postoperative consciousness recovery time. The intracranial hematoma volume was calculated using the coniglobus formula (v = ½ × a × b × c) based on the preoperative CT scan parameters. After implanting the ICP probe, ICP values were continuously recorded by the nursing staff every 4 h for 5 days. The recorded postoperative ICP values included ICP values (point and mean values), initial postoperative ICP value, and the duration for which the ICP values fluctuated. Under normal conditions, ICP in the lying position ranges from 7 to 15 mmHg. An ICP exceeding 20 mmHg can adversely affect the prognosis of patients with cerebral hemorrhage and brain trauma. When ICP exceeds 20 mmHg, it was represented by DICP20 mmHg × 4 h as a measure of the dose, reflecting the fluctuation and duration of ICP over a period of time. This parameter distinctly demonstrates an increased amplitude of ICP compared to that by conventional measurements. When the ICP was greater than 20 mmHg, the mannitol dosage was recorded. The short-term outcomes were evaluated based on the length of hospitalization and postoperative consciousness recovery time. The recovery of consciousness after surgery was assessed based on the following criteria: the ability to (1) open and close eyes and perform other eye movements as instructed and (2) perform limb activities.

### Statistical analysis

SPSS software (version 22.0; IBM, Armonk, NY, USA) was used for data entry and statistical analyses, and GraphPad Prism 8.0 was used for image processing. After confirming the distribution, Student’s unpaired t-test was used for intergroup data that conformed to a normal distribution, and the Mann-Whitney U test was used for intergroup data that conformed to a non-normal distribution. The quantitative data are presented as the mean ± standard deviation, whereas the qualitative data are presented in terms of frequency or percentage (%), and comparisons between groups were performed using the χ2 or Fisher’s exact test. A simple linear regression model was used to evaluate the trends in mean ICP values at various time points. The correlations of the ICP values on the first postoperative day with the postoperative consciousness recovery time and that of the mean ICP values with the length of hospitalization were determined via linear regression and Pearson correlation analyses, respectively. Statistical significance was set at p < 0.05. significance. *p < 0.05, ** p < 0.01, *** p < 0.001, **** p < 0.0001, ns (not significant) p > 0.05.

## Results

### Comparison of the baseline data of the two groups

Significant differences were observed in the postoperative initial ICP, ICP on the first postoperative day, mean ICP, DICP20 mmHg × 4 h, postoperative consciousness recovery time, and the length of hospitalization (p < 0.05). Age, sex, GCS score at admission, hematoma volume, and hematoma clearance rate were not significantly different between the groups (p > 0.05) (Table 1).

**Table 1:** Baseline information and the comparison of the short-term outcome between the two groups

Variable	TIA	TCA	Current Result	*p*
Age (mean ± standard deviation, years)	56.03 ± 7.55	55.40 ± 7.39	*t* = -0.374	0.787
Sex (male/female, patient)	23/17	25/15	*χ*^*2*^ = 0.208	0.648
GCS score at admission (mean ± standard deviation, points)	7.45 ± 1.95	7.55 ± 2.01	*t* = -0.226	0.706
Hematoma volume (mean ± standard deviation, ml)	42.11 ± 4.78	43.72 ± 4.94	*t* = -1.488	0.684
Hematoma clearance rate (mean ± standard deviation, %)	94.33 ± 2.88	93.98 ± 2.63	*t* = 0.576	0.312
Postoperative initial ICP value (mean ± standard deviation, mmHg)	4.28 ± 2.95	5.70 ± 3.07	*t* = 2.115	0.038
ICP value on the first postoperative day (mean ± standard deviation, mmHg)	6.72 ± 1.81	8.40 ± 2.39	*t* = 3.549	0.001
Mean ICP (mean ± standard deviation, mmHg)	11.82 ± 2.37	13.47 ± 2.86	*t* = 2.811	0.006
DICP20 mmHg × 4 h (median (interquartile range))	0(0,392)	80(0,709)	*Z* = -2.059	0.039
Postoperative consciousness recovery time (mean ± standard deviation, h)	12.30 ± 3.94	19.33 ± 7.62	*t* = 5.182	0.0001
The length of hospitalization (mean ± standard deviation, day)	18.25 ± 3.54	20.13 ± 4.05	*t* = 2.203	0.031

Mean ICP value is the average of postoperative ICP on 5 days

### Comparison of the postoperative initial ICP values, ICP values on the first postoperative day, ICP values on the third postoperative day, and mean ICP values between the two groups

**Fig. 1:**
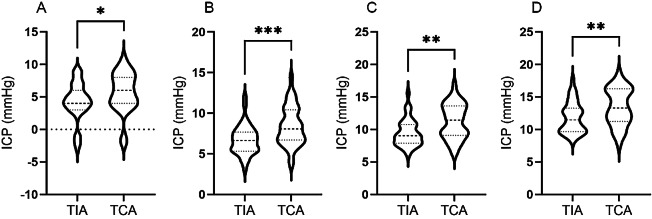
Analysis of the distribution and comparison of the postoperative ICP value between the two groups. (**A**) Postoperative initial ICP value; (**B**) ICP value on the first postoperative day; (**C**) ICP value for the three postoperative days; (**D**) Mean ICP value

Compared with that in the TCA group, the postoperative initial ICP decreased more significantly in the TIA group (p < 0.05; Fig. 1A). The postoperative ICP gradually increased after surgery; however, at 1, 3, and 5 days after surgery, the ICP in the TIA group was lower than that in the TCA group (p < 0.05; Fig. 1B, C, and D).

### Comparison of the trends in postoperative ICP values at various time points

**Fig. 2:**
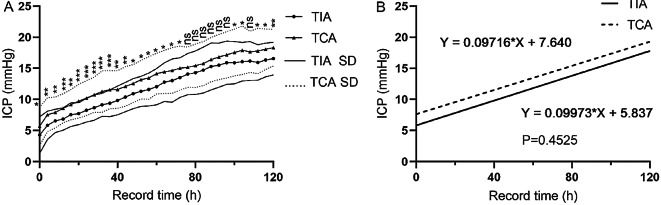
Comparison of the postoperative ICP values and the postoperative ICP values variation trends at various time points. (**A**) Compared the mean ICP values at various time points. (**B**) Compared the postoperative ICP values variation trends at various time points by the linear regression

The ICP values in the TIA group were significantly lower than those in the TIA group at all time points, except at the 76 to 86 and 92 time points (p < 0.05; Fig. 2A). Linear regression analysis of postoperative ICP values over time revealed no significant differences (p > 0.05; Fig. 2B).

### Comparison of the DICP20 mmHg × 4 h values, mannitol utilization rates, and mannitol dosages between the groups

**Fig. 3:**
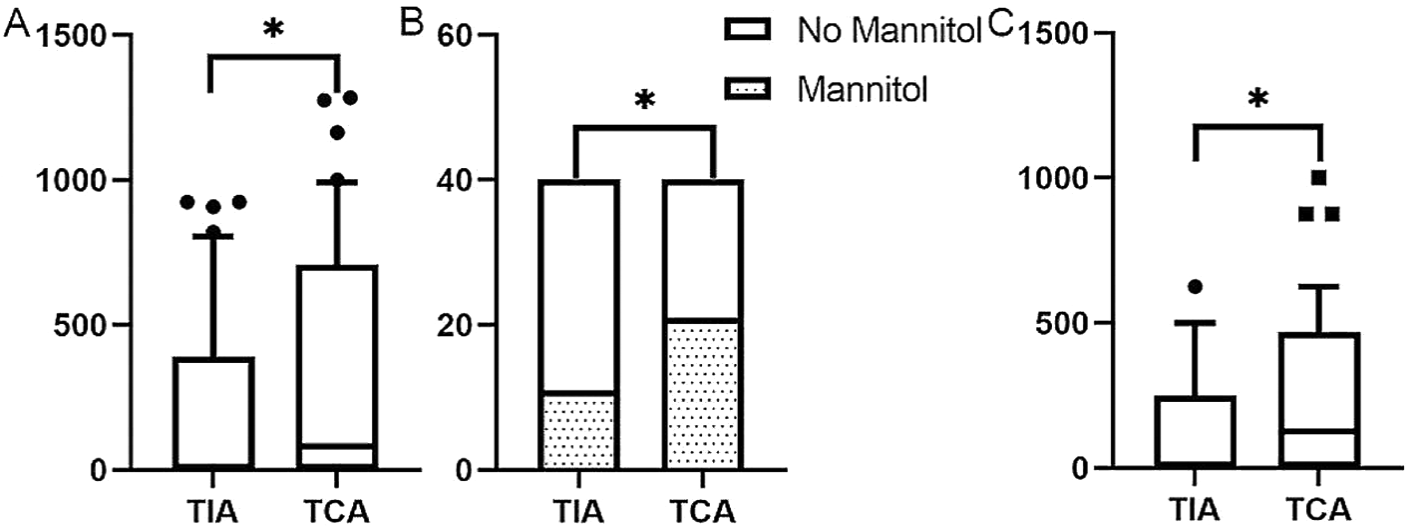
Comparison of the DICP20 mmHg × 4 h, mannitol utilization rate and mannitol dosage between the groups. (**A**) Comparison of the DICP20 mmHg × 4 h date dispersion. (**B**) The mannitol utilization rate. (**C**) The mannitol dosage (ml)

The DICP20 mmHg × 4 h values and mannitol dosages (mL), which did not correspond to a normal distribution, are expressed as boxes and whiskers (plot: 10th–90th percentile) and indicate a significantly greater degree of data dispersion in the TCA group than in the TIA group (p < 0.05; Fig. 3A and C). The frequency of DICP ≥ 20 mmHg was greater, and the utilization rate and dosage of mannitol were greater in the TCA group than those in the TIA group (p < 0.05; Fig. 3).

### Correlation between ICP values on the first postoperative day and postoperative consciousness recovery times

**Fig. 4:**
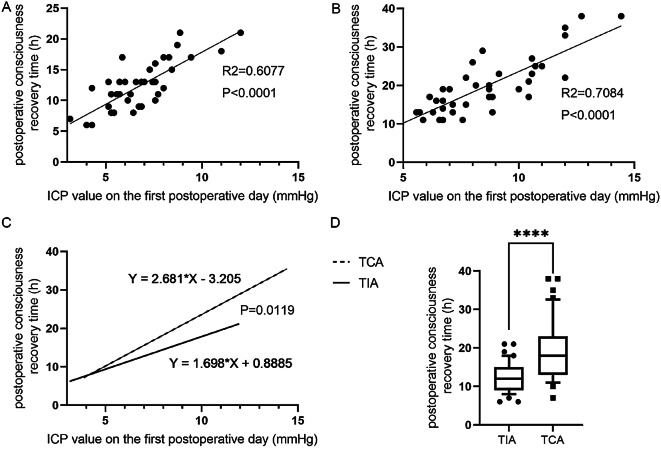
Correlation between postoperative ICP values and postoperative consciousness recovery times. (**A**) Correlation between the postoperative consciousness recovery times and the ICP values on the first postoperative day in the TIA group. (**B**) Correlation between the postoperative consciousness recovery times and the ICP values on the first postoperative day in the TCA group. (**C**) Comparison of the slope of the postoperative consciousness recovery times and the ICP values on the first postoperative day between the two groups. (**D**) Comparison of the postoperative consciousness recovery times between the two groups

Postoperative consciousness times are expressed as boxes and whiskers (plot: 10th–90th percentile) and were significantly different between the two groups (p < 0.0001; Fig. 4D). The postoperative consciousness time was positively correlated with ICP on the first postoperative day, and the correlation between the two groups was significant (p < 0.05; Fig. 4A, B, and C).

### Correlations between mean ICP values and hospitalization duration

**Fig. 5:**
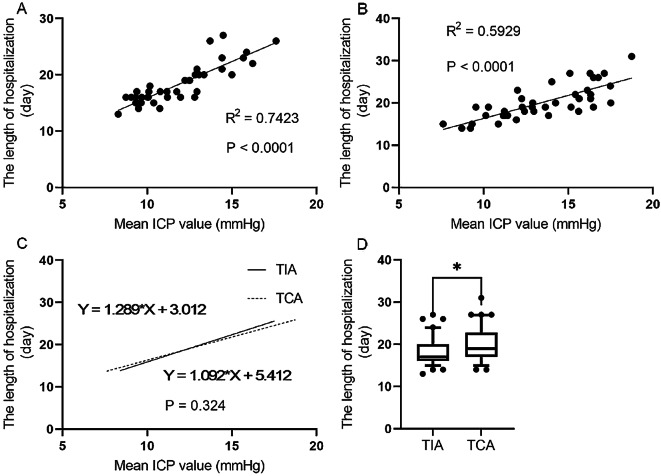
Correlation between mean ICP values and the length of hospitalization. (**A**) Correlation between the length of hospitalization and the mean ICP values in the TIA group. (**B**) Correlation between the length of hospitalization and the mean ICP values the TCA group. (**C**) Comparison of the slope of the length of hospitalization and the mean ICP values between the two groups. (**D**) Comparison of the length of hospitalization between the two groups

The length of hospitalization was significantly different between the two groups (p < 0.0001; Fig. 5D) and are expressed as boxes and whiskers (plot: 10th-90th percentile). The length of hospitalization was positively correlated with the mean ICP value, and there was no significant difference in the length of hospitalization between the two groups (p < 0.05; Fig. 5A, B, and C).

## Discussion

In patients with HICH, primary brain injury results from the mass effect of the hematoma, which is closely related to the volume and location of the hematoma. Peri-hematoma edema is the main source of secondary injury. The preoperative ICP increased mainly due to the mass effect of the hematoma and peri-hematoma edema, and the postoperative ICP increased mainly due to the rebuilding of intracerebral circulation and consequent congestion, brain edema, and re-hemorrhage [16]. In clinical practice, patients with HICH after surgery are in a state of coma, under sedation or muscle relaxant treatments; therefore, early clinical symptoms are not typical and could be ignored. Thus, once the clinical symptoms appear, the patient’s condition deteriorates sharply and the optimal intervention timing is missed. Currently, ICP monitoring can be quickly and accurately recorded, and postoperative dynamic monitoring of ICP can reflect intracranial conditions, which is particularly important in clinical work and is the key to managing postoperative treatment in a timely manner, beneficial for saving patients’ lives and improving their functional outcomes.

Surgical evacuation of an intracerebral hematoma is based on the theory of reducing mass effects, thereby decreasing ICP, improving cerebral blood flow, and reducing the release of toxic decomposition products by the clot. The possible negative side effects of this procedure include intracranial infection and additional trauma caused by the surgical procedure itself, possibly increasing the risk of re-hemorrhage by eliminating the compression of the hematoma [17]. Both TIA and TCA are minimally invasive surgical approaches commonly used by neurosurgeons and have the advantages of minimal invasiveness, good recovery, low mortality rates, and few complications. Unlike decompression craniotomy, decompression of the TIA or TCA is limited. Continuous and severe increases in ICP can lead to cerebrovascular circulation disorders and decreased cerebral perfusion volume, which can quickly result in ischemia and edema of the brain tissue. Although surgical treatment can eliminate hematomas to some extent and reduce ICP, the ICP of patients remain high during the early postoperative period, with large fluctuations in the ICP parameters [18]. Therefore, the implantation of ICP probes can help neurosurgeons detect abnormal increases in ICP in a timely manner and implement timely intervention measures.

Wu et al. [19]demonstrated that postoperative initial ICP is a good indicator of the short-term prognosis of patients with traumatic brain injury. This study revealed a significant reduction in the postoperative initial ICP value in the TIA group compared to that in the TCA group, which could be related to the ability of TIA to separate the Sylvian fissure, open the Sylvian fissure cistern, release cerebrospinal fluid, and sufficiently reduce ICP [20].

Mean ICP is closely related to postoperative changes in conditions and mannitol dose, which can improve patient prognosis and shorten hospitalization. In our study, the mean ICP was lower in the TIA group than in the TCA group. Moreover, repeated measurements in multiple groups revealed a significant difference in the trend of postoperative ICP over time, indicating that TIA could better improve postoperative ICP than did TCA, in patients with HICH; however, TIA or TCA alone could not change the trend of postoperative ICP. This difference could be because the Sylvian fissure area is a natural space in the brain, and its anatomical characteristics indicate that most hematomas are concentrated in the Sylvian fissure area. For TIA, only a part of the insular lobe must be cut to reach the hematoma and reduce damage to the cerebral cortex. TIA can also open the basal and Sylvian fissure cisterns and relieve the traction effect on the frontotemporal lobe after the release of cerebrospinal fluid. These findings indicate that TIA causes less damage to the brain tissue than TCA does, and the degree of postoperative brain edema is less severe. The frequency and dosage of mannitol in the TIA group were significantly lower than those in the control group, thus alleviating damage to renal function [21]. However, in the macroscopic comparison, we found that the changes in postoperative ICP did not significantly differ between the two groups over time, which may be related to technical problems that may have occurred during TIA surgery. TIA mainly requires the separation of the arachnoid membranes, lateral fissured veins, and branches of the middle cerebral artery around the lateral fissure cleat. The arteries were well protected during surgery. However, protecting the veins is difficult, and damage to these veins can easily occur, leading to venous cerebral infarction, which increases the probability of brain edema and ICP. TIA can effectively expose the lentiform artery, which often causes cerebral hemorrhage in the basal ganglia, facilitate electrocoagulation and hemostasis, and reduce the probability of a second operation due to rebleeding after surgery [22]. More than a decade ago, a study on TIA demonstrated that the TIA approach was superior to the TCA approach for reducing mortality and improving long-term outcomes and established it as the recommended approach for the treatment of intracerebral hematoma [23].

The length of hospitalization differed between the two groups and was shorter in the TIA group. The length of hospitalization was positively correlated with mean ICP, and there was no significant difference in the trends of the length of hospitalization between the two groups. We presume that TCA requires an incision in the temporal lobe cortex and that the pathway through the superior or middle temporal gyrus may damage the language center. Part of the frontal and temporal lobe brain tissues may be pulled to a greater extent, aggravating brain damage and edema, thus increasing the degree of increased ICP, resulting in a decrease in the prognosis of the patient compared with that during an ideal condition, along with the lengthening of hospitalization.

In our study, a DICP20 mmHg × 4 h was used to indicate the extent of changes occurring to ICP greater than the threshold of 20 mmHg over a period of time in the two groups, which could indicate the degree of brain injury and prognosis. The results of this study showed that the discreteness of DICP20 mmHg × 4 h in the TIA group was significantly lower than that in the TCA group, indicating that postoperative brain injury was less severe and that the prognosis was better in the TIA group than in the control group. ​.

​Patients with HICH have severe impairment of consciousness, resulting in difficulty swallowing, reduced cough reflexes, increased risk of aspiration and pulmonary infections, and difficulty discharging tracheal sputum. Early recovery of consciousness results in a short bed rest period, reduces the occurrence of solid pneumonia, increases the patient’s functional recovery time, and indicates good prognosis [24]. This study demonstrated a positive correlation between postoperative conscious recovery time, length of hospitalization, and postoperative ICP values, suggesting that monitoring the changes in postoperative ICP are important for postoperative management and patient recovery.

## Conclusion

In conclusion, our study suggests that TIA is more effective than TCA in improving the short-term outcomes of patients with basal ganglia hematoma volumes ranging from 30 to 50 mL. This was evidenced by the comparison of postoperative ICP parameters. Furthermore, these findings emphasize the importance of effectively managing ICP levels following surgical intervention to improve the postoperative management of patients with HICH.

## Data Availability

The datasets generated and/or analysed during the current study are not publicly available due to the protection of patients’ privacy but are available from the corresponding author on reasonable request.
